# Moving From Peripheral Project to Integrated Governance: Developing System Sustainability in Excellence Reporting

**DOI:** 10.1192/bjo.2022.338

**Published:** 2022-06-20

**Authors:** Jessica Scott, Jennifer Ledger, Helen Smith

**Affiliations:** Devon Partnership NHS Trust, Exeter, United Kingdom

## Abstract

**Aims:**

There has been increasing recognition that healthy cultures within NHS organisations are key to delivering high-quality, safe care (King's Fund). A focus towards developing systems which recognise and learn from excellence has been shown to improve services’ safety and contribute to staff's morale (Kelly *et al.* 2016). In 2019 Secure Services at Devon Partnership NHS Trust (DPT) developed an Excellence reporting system. Once successfully piloted, the intention was to extend to other departments before expanding to the entire Trust. Our aims initially were SMART: for 13 reports per week in Secure services and 8 in Perinatal (a smaller team). As we expanded the aim became qualitative: for a system to be embedded so staff could as readily and instinctively report Excellence as they could an error.

**Methods:**

We developed our Theory of Change using Deming's theory of profound knowledge, ran a series of PDSAs, and introduced an Excellence system. We engaged early adopters, sent hand-written cards and shared data widely.

Learning included understanding setting up the system, and the importance of a team rather than an individual holding the system. We took this forward to bring the system to Perinatal. We continued to run PDSAs, then ran monthly trust-wide meetings providing space to learn from other directorates.

**Results:**

Staff were initially excited, reports submitted, feedback good, then a plateau and slump.

Something was stopping the system perpetuating. When staff received timely thanks, and others heard about it, staff would go on to promote excellence. However, this was not possible without sufficient admin resources.

In early 2021 we changed tact and approached the top: we presented data to Directors who recognised the value and agreed to support. We then set about publicising the system, and demonstrating at trust-wide meetings.

By July 2021 we saw 10 reports per week in the Specialist Directorate.

By early 2022 reports were being inputted from staff across all directorates and our monthly meetings began to focus on sharing the learning.

**Conclusion:**

We recognised the system's potential impact on safety and staff morale but struggled to sustain the system and support dwindled when staff were stretched.

After approaching leaders, then allocated resources, it allowed for more success. However, it is not *yet* fully embedded in our Trust's culture.

A lot of our work happened during COVID-19 and despite challenges there has been a new-found flexibility to innovate, greater ease to negotiate, and instigate change.

